# Deciphering miRNA–Target Relationships to Understand miRNA-Mediated Carcinogenesis

**DOI:** 10.3390/cancers13102415

**Published:** 2021-05-17

**Authors:** Alfons Navarro

**Affiliations:** 1 Molecular Oncology and Embryology Laboratory, Human Anatomy Unit, Faculty of Medicine and Health Sciences, University of Barcelona, IDIBAPS, 08036 Barcelona, Spain; anavarroponz@ub.edu; Tel.: +34-934-021-903; 2 Thoracic Oncology Unit, Hospital Clinic, 08036 Barcelona, Spain

We now accept that the non-coding part of the genome is essential for fine-tuning most cellular functions, and that its deregulation drives carcinogenesis [1]. However, the first link between non-coding RNAs and cancer was identified only 16 years ago, where the microRNAs (miRNAs) miR-15/16 were identified as tumor suppressors in CLL by Croce’s group [2]. From that stepping stone multiple types of miRNAs first, and other non-coding RNAs later have been identified and related to all stages of tumor development, from tumor initiation to dissemination during the metastasis process [3]. Now the non-coding RNAs group has been so enlarged that people classify them in two main groups according to their size, where those of less than 200 nucleotides are call small non-coding RNAs, in contrast to long non-coding RNAs. The best studied non-coding RNA sequences of our genome, in part because they were the first formally related to cancer, are miRNAs, which belong to the small non-coding RNA group with a sequence length of 15–25 nt. The first miRNA, lin-4, was discovered in 1993 by V. Ambros’ group in *C. elegans* [4], but it took eight years to discover the second miRNA, let-7a, which really boosted miRNA discovery because in contrast to lin-4, homologous sequences of let-7a were discovered in most species including humans [5]. Since the finding of let-7a, the identification of new miRNAs has increased considerably. In fact, as of 2021, we know 2693 mature miRNAs in humans (according to miRBase v22.1). Several functions have been described for miRNAs (reviewed in [6]), but the most relevant one is the posttranscriptional regulation of protein-coding genes by binding to target mRNAs in a sequence-dependent way and inhibiting its translation. Therefore, to identify the functions of the dysregulated miRNAs, it is necessary to decipher their target genes, their specific targetome. But this task becomes complex, because each miRNA can target thousands of mRNAs and at the same time one mRNA is targeted by multiple different miRNAs. To increase the complexity, we know that the miRNA–target interactions depend on the cellular context and the specific associated transcriptome, which generate a cell type-dependent targetome for each miRNA that is also variable according to tumor types. Thousands of targets have been predicted for each miRNA using different bioinformatic algorithms such as TargetScan, miRanda or Pictar, which are mainly based in the identification of miRNA–target sequence complementarities, and a high number of them have been experimentally validated. However, an important gap exists yet between the number of pathological identified miRNAs and their real targets, since their complete targetome remains to be elucidated [7]. Recently, the miRNA–target network became more entangled with the addition of other non-coding RNAs, such as long non-coding RNAs or circRNAs, and with the fact that miRNAs not only regulate the transcriptome of the parental cell but also other cell types since they can be released outside through extracellular vesicles acting on other cellular transcriptomes different from the parental cell.

In this Special Issue of *Cancers* called “Role of miRNAs in Cancer—Analysis of Their Targetome” we collected 14 articles (5 original Articles, 1 Communication, 7 reviews and 1 Perspective). In the following lines I am going to summarize the main results of the articles included in the Special Issue. The original articles included move from the study of the role of individual miRNAs and their targets in different tumors, to the identification of new diagnostic/prognostic biomarkers in tissue and liquid biopsy.

Hunter et al. [8] studied the role of two *COX-2*-activated miRNAs, miR-526b and miR-655, in the control of angiogenesis and lymphomagenesis in an estrogen receptor positive breast cancer model. In the MCF7 cell line, these miRNAs by targeting *PTEN* induced *HIF1A*, which increased *VEGF* secretion. Moreover, the overexpression of these miRNAs enhanced the expression of *VEGFA/C/D*, *COX-2* and *LYVE1*. When HUVEC cells were cultured with the supernatant of overexpressing cells, VEGF and EP4 receptors augmented, and higher migration and higher tube formation was observed in HUVEC cells. In contrast, the use of *COX-2* inhibitors and *EP* antagonists reversed the phenotype. They also observed a correlation in patient samples between these miRNAs and angiogenesis and lymphomagenesis markers.

Borkowska et al. [9] studied the potential role as diagnostic biomarker of eight selected miRNAs (miR-10a, miR-20a, miR-21, miR-103, miR-130b, miR-145, miR-182 and miR-205) in 55 patients with bladder cancer and 30 controls. They concluded that the best diagnostic signature included miR-20a, miR-205 and miR-145, which discriminated bladder cancer from healthy controls.

Quin et al. [10] generated a sophisticated prognostic score in clear cell renal carcinoma patients, combining miRNAs and their target genes (TCGA data). The miRNA analysis initially identified eight miRNAs impacting patient outcome. The correlation with the expression data allowed the identification of mRNA signatures also impacting prognosis that explained the role of the identified miRNAs. To increase the efficiency of the miRNA score, the authors identified transcription factors associated with the miRNA signature and generated a combined score including miR-365b-3p, miR-223-3p, miR-1269a, miR-144-5p, miR-183-5p, miR-335-3p, *TFAP2A*, *KLF5*, *IRF1*, *MYC* and *IKZF1* which had the highest impact in patient survival. They concluded that transcription factors and miRNAs can cooperatively regulate oncogenesis and impact prognosis in clear cell renal carcinoma patients.

Niu et al. [11] by small RNAseq identified miR-378a-3p as an upregulated miRNA impacting proliferation in Burkitt lymphoma cell lines. After validating its overexpression in patient tissues, they performed targetome analysis by Ago2-RIP-Chip. The bioinformatic analysis of the results allowed them to identify 63 potential targets when they inhibited the miRNA in ST486 cells, and 20 targets when they overexpressed it. The authors focused on *MYCBP*, *CISH*, *BCR*, *TUB1C*, *FOXP1*, *MNT*, *IRAK4* and the lncRNA *JPX* for validation with luciferase assays finally confirming *FOXP1*, *MNT*, *IRAK4* and the lncRNA *JPX* as real miR-378-3p targets.

Cuscino et al. [12] identified eight novel miRNAs in osteosarcoma by analyzing the cellular and exosomal RNA from the cell lines SAOS-2, MG-63 and U-2 OS. The validation in tissue and plasma samples from osteosarcoma patients showed that seven miRNAs were detected in all samples, and five where significantly upregulated in plasma samples. The in silico analysis of the miRNA–targets revealed several KEGG pathways linked to cancer, suggesting that the novel miRNAs identified could have a role in osteosarcoma pathogenesis.

Nguyen et al. [13] made a combined article that simultaneously reviewed the literature and at the same time provided their own data on blood miRNAs as biomarkers for radiotherapy response in pancreatic cancer. Using mice harboring pancreatic tumors, they obtained by small RNAseq a miRNA signature associated with the presence of this tumor. Then, they treated the mice with radiotherapy (a single 5Gy dose) and at 24 h plasma was collected for miRNA analysis. A miRNA signature that included 20 downregulated miRNAs and upregulated one (miR-184) was identified. The role of these miRNAs and their targets were revised showing that several of them have been previously related to radioresistance in other tumor models, but only a few of them have been previously associated with pancreatic cancer radioresistance.

The review articles included in the Special Issue cover various topics from the known miRNA targetome in specific tumors, including glioblastoma, thyroid or adrenocortical cancer, to the analysis of miRNAs involved in the metastasis process in general or focusing in the targetome of epithelial mesenchymal transition (EMT) genes such as SNAIL or the involvement of miRNAs in the crosstalk between tumor and immune system cells. Moreover, specific targetomes such as miR-361 or viral-associated miRNA targetome have been explored.

Xu et al. [14] concentrated on a unique miRNA, miR-361, which can be considered a tumor suppressor miRNA because its targets are mostly oncogenes. They summarized the main reasons for miR-361 downregulation, which include DNA hypermethylation, transcriptional inhibition, sponging by lncRNAs and gene deletion. The cellular and extracellular targetome of this miRNA explains the aggressive tumor phenotype observed when the miRNA is lost and its potential utility as diagnostic, prognostic or therapeutic biomarker.

Sole et al. [15] nicely summarized the miRNAs involved in the metastasis process. They organized the miRNAs into three groups: metastasis-promoting miRNAs, metastasis-suppressing miRNAs, and metastasis associated circulating blood miRNAs. They exhaustively listed the miRNAs associated with metastasis in different tumors together with their known targets.

Skrzypek et al. [16] also examined metastasis but centering on the *SNAIL* transcription factor, which is involved in EMT regulation and metastasis. The authors reviewed not only miRNAs, but also lncRNAs and circRNAs that either regulate *SNAIL* levels or that are regulated by *SNAIL*.

Gallo et al. [17] examined the role of viral (mainly herpesvirus) miRNAs and their cellular targets in the tumorigenesis process. The article listed the known miRNAs from EBV, HPV, KSHV/HHV8, HBV and MCPyV and their cellular targetomes involved in both the own viral cycle control and the induction of tumorigeneses.

In glioblastoma multiforme, the methylation status of the *MGMT* gene classifies patients into unmethylated and methylated, and the last group is treated with totemozolomide-based chemotherapy. Kirstein et al. [18] discussed the potential role of miRNAs targeting *MGMT* as therapeutic tools for unmethylated patients, which are resistance totemozolomide-based chemotherapy. They examined whether miRNAs inhibiting *MGMT* could enhance response to this line of treatment.

Chehade et al. [19] reviewed miRNAs associated with adrenocortical cancer, which is a rare but aggressive malignancy. They revised from differentially expressed miRNAs in tumor tissue to differentially expressed circulating miRNAs, and their utility as diagnostic/prognostic biomarkers. Moreover, they revised the known targetome of adrenocortical cancer miRNAs focusing on relevant disease-associated pathways such a p53 pathway, mTOR or Wnt/B-catenin.

Tabatabaeian et al. [20] presented a systematic review on miRNAs, lncRNAs and circRNAs in thyroid cancer. They summarized the most relevant non-coding RNAs involved in the tumorigenesis process and their role as diagnostic, prognostic or therapeutic biomarkers. Of note, they highlighted the list of ongoing clinical trials which includes some of these non-coding RNA biomarkers.

Lastly, Cho et al. [21] briefly reviewed the miRNA targetome involved in the interaction between tumor and immune cells, stressing the miRNAs responsible for inhibiting the anti-tumor immune response. They focused on two main groups: miRNAs released in exosomes and participating in the crosstalk between tumor cells, mesenchymal cells and immune cells (macrophages, CD4+ T cells or CD8+ T cells); and cellular miRNAs regulating the levels of immunomodulatory proteins such as CD47, IDO1 or PD-L1 in the tumor cell. Moreover, the authors discussed the main techniques used for study of the miRNA targetome such as CLIP-seq or CLASH, that are necessary to fully understand the role of miRNAs in tumorigenesis.

In summary, this Special Issue includes miRNA-related articles that add new information to the miRNA–target interaction in different tumor models and review some of the most recent information on the tumorigenic targetome. However, more efforts are still needed to decipher the critical targets—both coding and non-coding RNAs—of the miRNAs involved in cancer to identify their contribution to the malignant transformation and metastasis process.
